# Early postoperative calcitonin-to-preoperative calcitonin ratio as a predictive marker for structural recurrence in sporadic medullary thyroid cancer: A retrospective study

**DOI:** 10.3389/fendo.2022.1094242

**Published:** 2022-12-16

**Authors:** Zan Jiao, Tong Wu, Mingjie Jiang, Shuxian Jiang, Ke Jiang, Jin Peng, Guangfeng Luo, Yongchao Yu, Weichao Chen, Ankui Yang

**Affiliations:** ^1^ Department of Head and Neck Surgery, Sun Yat-sen University Cancer Center, Guangzhou, China; ^2^ State Key Laboratory of Oncology in Southern China, Collaborative Innovation Center for Cancer Medicine, Guangzhou, China; ^3^ Department of Plastic Surgery, The First Affiliated Hospital, Jinan University, Guangzhou, China

**Keywords:** medullary thyroid cancer, prognosis, calcitonin, CR, sporadic disease

## Abstract

**Background:**

Calcitonin (Ctn) is widely used as a marker in the diagnosis, prognosis, and postoperative follow-up of patients with medullary thyroid carcinoma (MTC). The prognostic value of postoperative calcitonin-to-preoperative calcitonin ratio (CR), reflecting the change in Ctn level of response to initial treatment, remains uncertain in long-term disease outcomes. This study aims to determine the cut-off value of CR for predicting structural recurrence and assess the prognostic role of CR in patients with MTC.

**Methods:**

We retrospectively reviewed patients with MTC in Sun Yat-sen University Cancer Center (SYSUCC) between 2000 and 2022. CR is defined as the ratio of postoperative Ctn level on the day of discharge divided by preoperative Ctn level. In order to determine the optimal cut-off value of CR, the receiver operating characteristic (ROC) analysis was performed. We evaluate the effect of CR on recurrence-free survival (RFS) by using the Kaplan-Meier method and Cox regression analysis. Then, a nomogram based on CR was constructed.

**Results:**

In total, 112 sporadic MTC patients were included in this study. The optimal cut-off value of CR that predicted disease recurrence was 0.125. Patients with CR≥0.125 showed significantly worse RFS than patients with CR <0.125, respectively (3-years RFS rate of 63.1 vs. 94.7%, 5-years RFS rate of 50.7 vs. 90.3%, P < 0.001). In the multivariate analysis, CR was the strongest independent predictor of structural recurrence (HR: 5.050, 95% CI: 2.247–11.349, *P <*0.001). Tumor size (HR: 1.321, 95% CI: 1.010–1.726, *P* =0.042), multifocality (HR: 2.258, 95% CI: 1.008–5.058, *P* =0.048) and metastasized lymph nodes (HR: 3.793, 95% CI: 1.617–8.897, *P <*0.001) were also independent predictors of structural recurrence. The uncorrected concordance index (c-index) of the nomogram was 0.827 (95% CI, 0.729-0.925) for RFS, and bias-corrected c-index were similar. As compared to TNM stage, the nomogram based on CR provided better discrimination accuracy.

**Conclusions:**

We demonstrate that CR is a strong prognostic marker to predict structural recurrence in patients with sporadic MTC. The nomogram incorporating CR provided useful prediction of RFS for patients with sporadic MTC to provide personalized treatment.

## 1 Introduction

Medullary thyroid carcinoma (MTC) arising from calcitonin-producing parafollicula is a rare neuroendocrine malignancy (1, 2). MTC accounting for only 1%–2% of all thyroid carcinomas, but leads to approximately 13% of thyroid cancer-related deaths, with a poorer clinical prognosis than papillary thyroid carcinoma (3, 4). Approximately 75% of cases occur sporadically, whereas the remaining 25% are associated with part of three inherited syndromes: familial MTC, multiple endocrine neoplasia 2A (MEN2A) or MEN2B (3, 5). Total thyroidectomy remains the most effective treatment leading to a cure for MTC, which is recommended by the American Thyroid Association (ATA) guidelines (3). However, biochemical cure (ie, normalization of serum calcitonin levels) is achieved in only 40%-60% patients following radical surgery (6, 7).

Calcitonin (Ctn), a 32-amino hormone, is secreted from parafollicular C cells of the thyroid gland (8). Ctn is a highly sensitive and specific tumor marker of MTC routinely used in diagnosis, prognostic assessment, and therapeutic follow-up (9, 10). Persistently high Ctn levels may indicate recurrent or residual disease, so it is significant to mandate regular serum Ctn monitoring after surgery (11, 12). Similarly, Ctn and carcinoembryonic antigen (CEA) doubling time have been considered to excellent predictors of progression during follow-up (11–13). Ctn doubling time less than 2 years can adequately predict tumor progression of MTC over time (12, 13). However, to calculate the Ctn doubling time, it requires a long-term follow-up with multiple detection and different detection methods of serum Ctn may reduce the clinical effectiveness over time.

Few studies have evaluated the change in Ctn level of response to initial treatment during the perioperative period. A study focusing on undetectable postoperative Ctn indicated that it is a prognostic factor of disease-free survival in MTC (14). Recently, biochemical response of Ctn to surgery was also recognized as a significant predictor of clinical outcome (15). Machens et al. showed that serum Ctn levels after initial surgery typically normalize within 1 week (16). Given the perioperative change of Ctn level is an objective dynamic indicator combining preoperative Ctn and early postoperative Ctn comprehensively, we sought to establish a novel marker called postoperative calcitonin-to-preoperative calcitonin ratio (CR). Thus, CR is calculated by dividing postoperative Ctn level on the day of discharge after surgery by preoperative Ctn, which seems to be a reliable marker to reflect the response of initial treatment in the early follow-up.

Herein, we used a cohort of 112 patients with sporadic MTC to investigate the role of CR as a prognostic biomarker of MTC for recurrence-free survival.

## 2 Methods and materials

### 2.1 Study cohort

**Figure 1:**
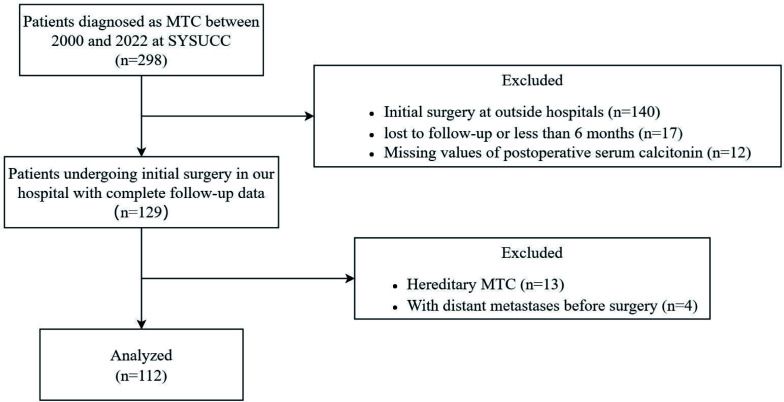
Flowchart of the patient selection process. MTC, medullary thyroid carcinoma; SYSUCC, Sun Yat-sen University Cancer Center.

We retrospectively reviewed patients who underwent thyroid surgery for MTC at Sun Yat-sen University Cancer Center (SYSUCC) between January 2000 and May 2022 (n=298). The exclusion criteria were as follows: (1) undergoing initial surgery at outside hospitals (n=140); (2) follow-up duration less than 6 months or lost to follow-up (n=17); (3) lack of postoperative serum calcitonin levels or postoperative imaging studies (n=12); (4) hereditary MTC patients (n=13); (5) patients with distant metastases before surgery (n=4), to avoid potential confounding factors. After exclusion, a total of 112 patients with confirmed MTC and adequate data were included in our study (**Figure 1**).

This study was approved by the Medical Ethics Committee of SYSUCC. Written informed consent was obtained from all patients in the hospital to offer related information.

### 2.2 Management and follow-up

Our institution follows the current guidelines for MTC treatment (3, 9). All patients underwent neck ultrasound (US) before operation to assess the primary lesions and the extent of lymph nodes. Total thyroidectomy with central lymph node neck dissection was performed in all patients, regardless of tumor size and the thresholds of various biochemical markers used in the MTC diagnosis, such as Ctn, CEA and procalcitonin (PCT) (17). Furthermore, patients with confirmed fine-needle aspiration biopsy evidence or preoperative suspicious imaging of lateral lymph node metastasis were treat with therapeutic lateral neck dissection. Standard pathological diagnoses of MTC were based on the ATA guidelines (3).

In the first 2 years, all patients were followed up every 6 months and then checked annually. The follow-up evaluation included regular measurements of serum Ctn, CEA and thyrotropin (TSH) as well as US. Patients with elevated or demonstrated rising postoperative serum Ctn levels underwent additional imaging studies to evaluate distant metastases, including neck CT, chest CT, and if distant metastasis is suspected, luoride-18 fluorodeoxyglucose integrated with positron emission tomography/CT (18F-FDG PET/CT) also should be carried out.

### 2.3 Outcomes

Disease status of MTC is currently based on clinical examination, Ctn, neck US, and additional imaging examinations. At final follow-up, patients were considered to disease-free if they had no evidence of structural disease and undetectable Ctn. Patients with detectable Ctn above the noramal range without structural disease were defined as biochemical persistent disease. If imaging examinations for patients with any structural disease, patients were considered as structural persistent disease. The primary endpoint was structural recurrence. Local and regional recurrences were confirmed by histopathology or cytology, while diagnosis of distant metastasis was based on imaging. Recurrence-free survival (RFS) was calculated from the time of initial surgery for MTC to structural recurrence or last follow-up.

### 2.4 CR calculation

Within single patients, the same Ctn assay was used to measure the change in postoperative Ctn level on the day of discharge compared with the preoperative Ctn level. Detection of Ctn was performed by chemiluminescence using the MAGKUMI 4000 System at the clinical laboratory of SYSUCC and normal range of Ctn was 0-18 pg/mL. These postoperative blood samples were collected at the third or fourth day after surgery from patients who had central lymph node dissection, and at the fifth or sixth day after lateral lymph node dissection. For the aim of the study, we establish a novel marker called CR. Then we divide postoperative Ctn level on the day of discharge by preoperative Ctn to calculate the CR value.

### 2.5 Statistical analysis

Data for continuous variables are expressed as mean (SD) or median and interquartile range (IQR) while data for categorical variables are expressed as absolute numbers and percentages. The Student’s t-test was used to compare continuous variables, and the Chi-square test or Fisher’s exact test was used to compare categorical variables. To investigate differences between CR and baseline characteristics, the optimal cut-off value of CR was determined by a Receiver operating characteristic (ROC) to categorize the groups. Kaplan-Meier curve with log-rank test was generated to assess RFS. A multivariable Cox proportional hazard model was constructed using stepwise selection method from the Akaike information criterion (AIC) with a univariable inclusion criterion of *P* < 0.05 to identify prognostic factors for disease recurrence. Then, we established a nomogram based on the multivariate COX regression model with endpoints of 3-year and 5-year RFS. Bootstrap method with 1000 resamples was used for internal verification of the nomogram. The accuracy of predictions was evaluated by estimating the model’s calibration, and discrimination was measured by the concordance index (C-index). ROC curves were used to compare the predicting ability of nomogram and TNM staging system by computing the area under the curve (AUC). All statistical tests were conducted using SPSS version 26.0 (IBM) and R version 4.1.2. P values < 0.05 were considered statistically significant for the statistical analyses.

## 3 Results

### 3.1 Baseline characteristics

As summarized in **Table 1**, a total of 112 patients were included in the study, including 56 men (50.0%) and 56 women (40.0%), with a mean age of 44.0 ± 14.1 years (range, 11-74 years) at initial surgery. Body mass index (BMI) at diagnosis was 22.80 ± 0.32 kg/m^2^. In the pathology reports, the median tumor size was 2.0 (IQR, 1.2-2.5) cm and the median number of metastasized lymph nodes was 2 (IQR, 0-9), with 27.7% having multifocal tumors, and 17.9% with extrathyroidal extension. According to the 8th edition of the American Joint Committee on Cancer (*AJCC*) TNM staging system, 29 (25.9%) patients were classified as stage I, 15 (13.4%) patients as stage II, 22 (19.6%) patients as stage III and 46 (41.1%) patients as stage IV. The median follow-up duration was 46.5 (IQR, 25.0-92.5) years.

**Table 1:** Characteristics of patients classified according to the CR.

Characteristics	Total	CR<0.125	CR≥0.125	*P*-value
	N=112	N=81	N=31	
**Age (years, mean ± SD)**	44.0 ± 14.1	44.1 ± 13.6	43.9 ± 15.6	0.963
**Sex (n, %)**				0.833
female	56 (50.00)	41 (50.6)	15 (48.4)	
male	56 (50.00)	40 (49.4)	16 (51.6)	
**BMI (kg/m^2^, mean ± SD)**	22.0 ± 3.49	21.98 ± 3.34	22.15 ± 3.90	0.819
**Tumor size (cm, median, IQR)**	2.0 (1.2-2.5)	2.0 (1.5-2.5)	1.6 (1.0-3.0)	0.634
**Metastasized lymph nodes (number, median, IQR)**	2 (0-9)	1 (0-7)	7 (1-15)	0.006
**Multifocality (n, %)**				0.253
No	81 (72.3)	61 (75.3)	20 (64.5)	
Yes	31 (27.7)	20 (24.7)	11 (35.5)	
**Extrathyroidal extension (n, %)**				0.003
No	92 (82.1)	72 (88.9)	20 (64.5)	
Yes	20 (17.9)	9 (11.1)	11 (35.5)	
**T stage (n, %)**				<0.001
T1	49 (43.8)	35 (43.2)	14 (45.2)	
T2	38 (33.9)	35 (43.2)	3 (9.7)	
T3	11 (9.8)	6 (7.4)	5 (16.1)	
T4	14 (12.5)	5 (6.2)	9 (29.0)	
**N stage (n, %)**				0.072
N0	44 (39.3)	36 (44.4)	8 (25.8)	
N1a	22 (19.6)	17 (21.0)	5 (16.1)	
N1b	46 (41.1)	28 (34.6)	18 (58.1)	
**AJCC 8th TNM stage (n, %)**				0.137
I	29 (25.9)	23 (28.4)	6 (19.3)	
II	15 (13.4)	13 (16.0)	2 (6.5)	
III	22 (19.6)	17 (21.0)	5 (16.1)	
IV	46 (41.1)	28 (34.6)	22 (58.1)	
**Follow-up duration (months, median, IQR)**	46.50 (25.0-92.5)	47.0 (28.0-94.0)	44.00 (19.0-81.5)	0.349
**Final disease status (n, %)**				<0.001
Disease-free	72 (64.3)	65 (80.3)	7 (22.6)	
Biochemical persistent disease	12 (10.7)	6 (7.4)	6 (19.3)	
Structural disease	28 (25.0)	10 (12.3)	18 (58.1)	

CR, postoperative calcitonin-to-preoperative calcitonin ratio; BMI, body mass index; AJCC, American Joint Cancer Committee; IQR, interquartile range; SD, standard deviation.

### 3.2 The association between CR and clinical characteristics

**Figure 2:**
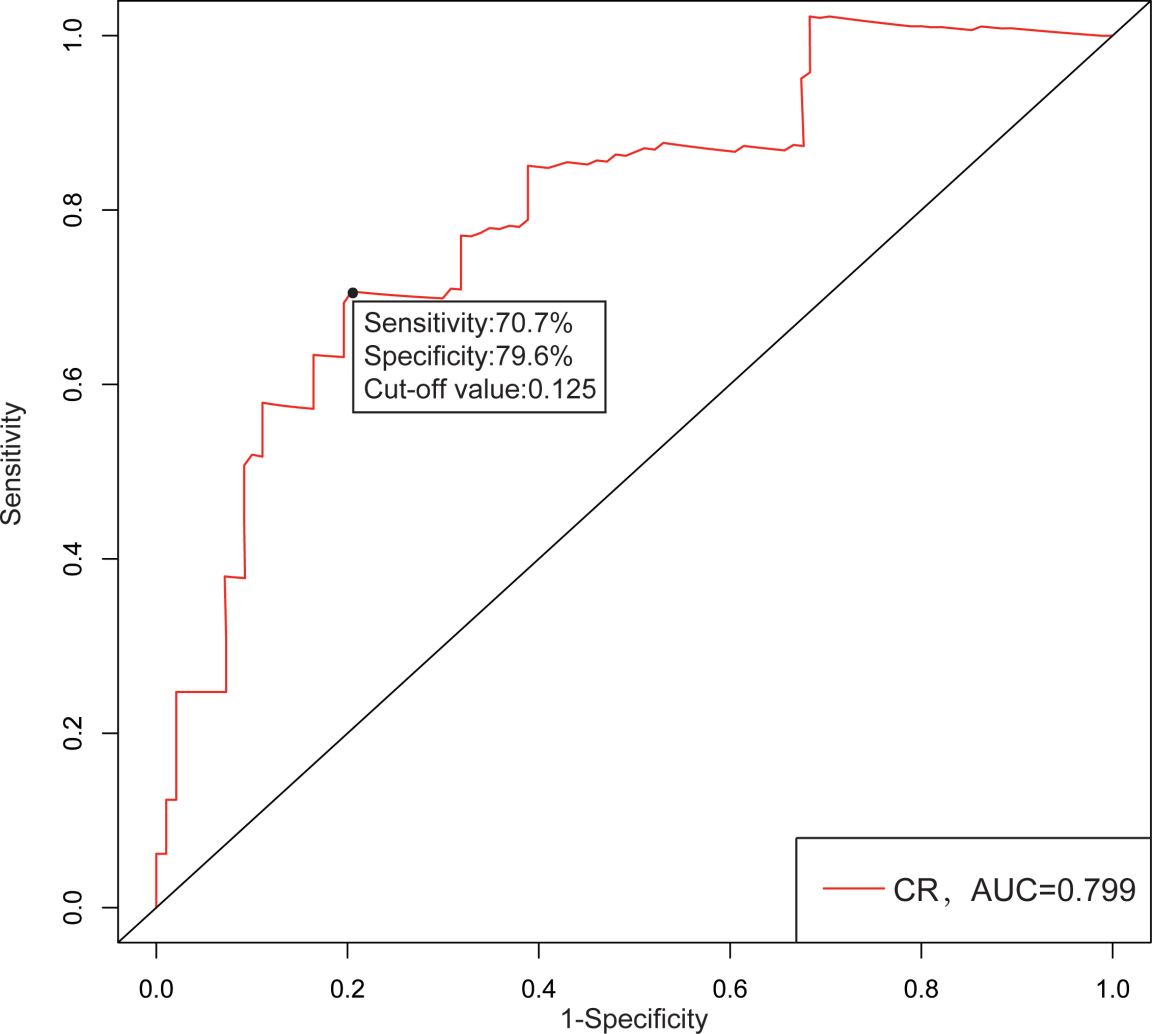
The receiver operating characteristic (ROC) analysis for CR cut-off value. CR, postoperative calcitonin-to-preoperative calcitonin ratio.

As calculated by the ROC curve with the 3-year RFS as an endpoint, the optimal cut-off value of CR was determined to be 0.125 and the AUC for CR was 0.799, respectively (**Figure 2**). Based on the cut-off value, all patients were further divided into two subgroups: CR<0.125 and CR≥0.125. The clinical and oncological features of the two subgroups were shown in **Table 1**. There were no differences between the two groups in age, sex, BMI, tumor size, multifocality, follow-up duration, N stage and AJCC 8^th^ TNM stage; however, patients with CR≥0.125 were significantly associated with more metastasized lymph nodes (*P* = 0.006), gross extrathyroidal extension (*P* = 0.003), higher T stage (*P* < 0.001) and worse final disease status (*P* < 0.001).

**Figure 3:**
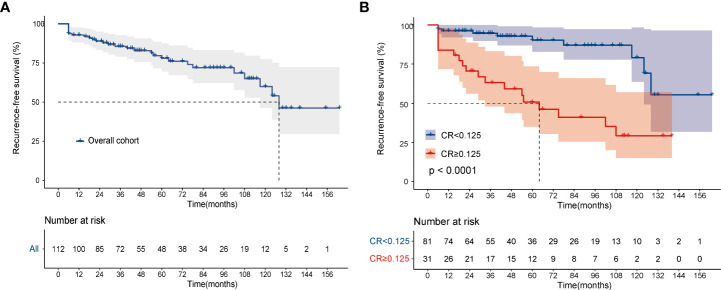
Kaplan-Meier curves for recurrence-free survival. **(A)** overall cohort; **(B)** according to CR. The log-rank test was used to compare curves. CR, postoperative calcitonin-to-preoperative calcitonin ratio.

Data on final disease status were available for 112 patients, 81 in the CR <0.125 group and 31 in the CR≥0.125 group. After a median follow-up of 47.0 (IQR, 28.0–94.0) months, 65 (80.3%) patients with CR <0.125 were disease-free, whereas 6 (7.4%) had persistent biochemical disease. 9 (11.1%) patients in the CR <0.125 group progressed to structural disease and only 1 (1.2%) died of MTC. In the CR≥0.125 group (n=31), after a median follow-up of 44.0 (IQR, 19.0–81.5) months, 7 patients (22.6%) were classified as disease-free, 6 (19.3%) as biochemical disease, 15 (48.4%) as structural disease, and 3 (9.7%) died of disease-related events. Then, the Kaplan‐Meier analysis showed that the 3- and 5‐year RFS of the overall cohort were 85.6% and 78.0% (**Figure 3A**), while patients in the CR≥0.125 group showed significantly worse RFS than those in the CR <0.125 group, respectively (3-years RFS of 63.1 vs. 94.7%, 5-years RFS of 50.7 vs. 90.3%, *P* < 0.001, **Figure 3B**). CR remained associated with a long-term recurrence-free status.

### 3.3 Factors predicting recurrence-free survival

**Table 2** shows the univariate and multivariable analysis of prognostic factors for RFS in MTC using Cox regression models. Univariate analysis revealed that larger tumor size (*P=*0.003), multifocality (*P=*0.007), gross extrathyroidal extension (*P=*0.002), higher T stage (T3-T4) (*P=*0.004), numbers of metastasized lymph nodes≥10 (*P*<0.001) and advanced TNM stage (III-IV) (*P=*0.003). Variables selected according to a univariable inclusion criterion of *P* < 0.05 were compared in a multivariate model by using a stepwise method. When all factors with *P* < 0.05 in the univariable analysis were included in the multivariate regression analysis, the AIC was 192.98. After adjustment of the model by using the stepwise selection method, three factors of gross extrathyroidal extension, T stage and TNM stage were removed, then the AIC of final model was 188.67. Finally, we constructed the multivariate Cox regression model (**Table 2**). In the final multivariable analysis, tumor size (HR: 1.321, 95% CI: 1.010–1.726, *P* =0.042), multifocality (HR: 2.258, 95% CI: 1.008–5.058, *P* =0.048), metastasized lymph nodes (HR: 3.793, 95% CI: 1.617–8.897, *P <*0.001) and CR (HR: 5.050, 95% CI: 2.247–11.349, *P <*0.001) were independent risk factors of structural recurrence.

**Table 2:** Univariate and multivariate Cox analysis for recurrence-free survival.

Variables	Univariable analysis		Multivariable analysis	
	HR (95% CI)	*P*-value	HR (95% CI)	*P*-value
**Age (years)**	0.973 (0.947-1.000)	0.051		
Sex				
female	1 (reference)			
male	1.137 (0.540-2.396)	0.735		
**BMI (kg/m^2^)**	1.293 (0.923-1.148)	0.603		
**Tumor size (cm)**	1.506 (1.155-1.964)	0.003	1.321 (1.010-1.726)	0.042
Multifocality				
No	1 (reference)		1 (reference)	
Yes	2.785 (1.318-5.885)	0.007	2.258 (1.008-5.058)	0.048
Extrathyroidal extension				
No	1 (reference)			
Yes	3.462 (1.589-7.543)	0.002		
T stage				
T1+T2	1 (reference)			
T3+T4	5.229 (2.364-11.564)	<0.001		
Metastasized lymph nodes				
<10	1 (reference)		1 (reference)	
≥10	5.778 (2.692-12.404)	<0.001	3.793 (1.617-8.897)	0.002
AJCC 8th TNM stage				
I+II	1 (reference)			
III+IV	3.860(1.462-10.194)	0.006		
CR				
<0.125	1 (reference)		1 (reference)	
≥0.125	5.224(2.404-11.352)	<0.001	5.050 (2.247-11.349)	<0.001

HR, hazard ratio; CI, confidence interval; BMI, body mass index; AJCC, American Joint Committee on Cancer; CR, postoperative calcitonin-to-preoperative calcitonin ratio.

### 3.3 Nomogram establishment and internal validation

**Figure 4:**
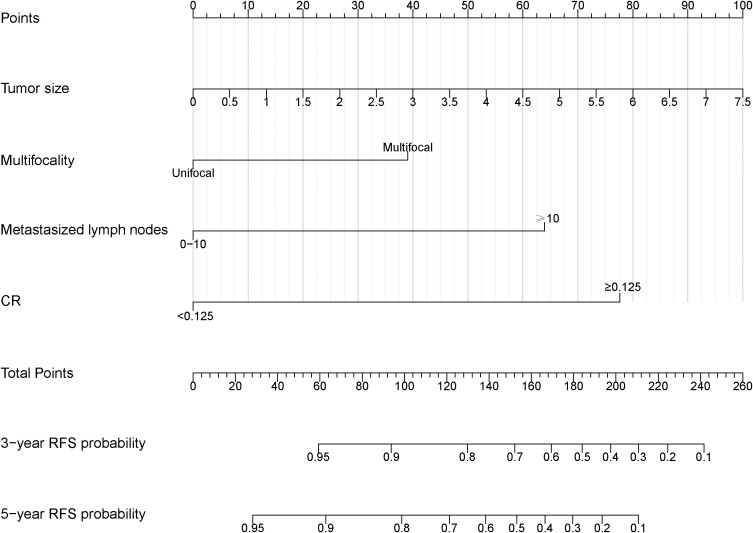
Nomogram to predict the probabilities of 3-year and 5-year RFS for MTC patients. The nomogram was constructed by tumor size, multifocality, metastasized lymph nodes and CR. RFS, recurrence-free survival. CR, postoperative calcitonin-to-preoperative calcitonin ratio.

**Figure 5:**
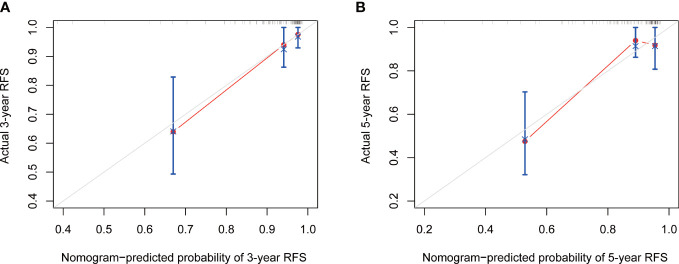
The calibration curve of nomogram for predicting 3-year **(A)** and 5-year **(B)** RFS. RFS, recurrence-free survival.

**Figure 6:**
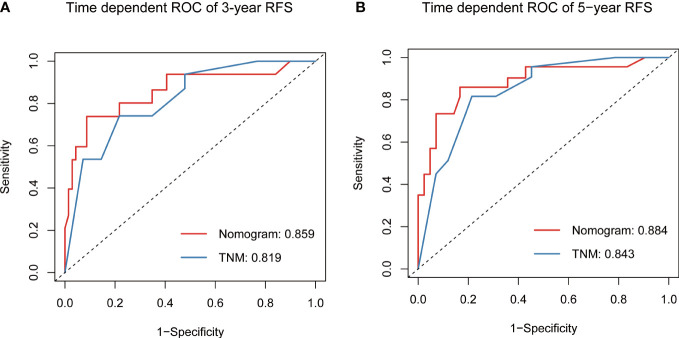
Predictive performance of nomogram compared with TNM stage for RFS at 3-year **(A)** and 5-year **(B)**, by time-dependent receiver operating characteristic (ROC) curve analysis. RFS, recurrence-free survival.

Based on the final multivariable model, we constructed a nomogram for RFS among these patients (**Figure 4**). The following four indicators were selected: tumor size, multifocality, metastasized lymph nodes and CR. The scores for each independent predictor were plotted and serially summed up to obtain the total score for predicting 3- and 5-year RFS. Model performance was validated for by measuring both discrimination and calibration. The C-index for the nomogram prediction model was 0.827 (95% CI, 0.729-0.925) and the bias corrected C-index generated by bootstrap validations was 0.834 (95% CI, 0.660-0.892), demonstrating a good predictive accuracy. The calibration curve (**Figures 5A, B**) adjusted by bootstrapping with 1,000 samples showed that the 3- and 5-year RFS predicted by the nomogram which showed a good degree of fit between the predictions and observations. Furthermore, the predictive ability of the nomogram was compared with the TNM staging system. The C-index of our nomogram was superior to that of the TNM staging system, which had a C-index for RFS of 0.802 (95% CI, 0.718-0.886). In the analysis of the 3- and 5-years RFS, the AUCs of the nomogram were 0.859 (95% CI, 0.742– 0.977) and 0.884 (95% CI, 0.789– 0.978), respectively, which were both higher than those of TNM staging system [0.804 (95% CI, 0.697–0.912) and 0.820 (95% CI, 0.717–0.922)] (**Figures 6A, B**), demonstrating that this nomogram can be used to determine the prognosis more accurately than the traditional TNM staging system.

## 4 Discussion

The optimal approach for follow-up of sporadic MTC patients remains controversial, particularly for those with biochemical incomplete responses. It was reported that only 27-42% of MTC patients were excellent response after initial surgical treatment, with a large proportion of patients having a biochemical incomplete response or structural incomplete response. While 26-51% biochemical incomplete patients revealed no disease progression at long-term follow-up, 32-37% had structurally persistent disease and even died from MTC (14, 18). It is crucial to identify which patients are more susceptible to structural recurrence, however, there are few reliable markers that can be used to predict postoperative outcomes.

Ctn is a serum biomarker that is routinely used in the diagnosis, and postoperative follow-up of patients with MTC. Current guidelines recommend measurement of Ctn in order to evaluate patient follow-up (3). A recent study showed that preoperative Ctn with a threshold of 309 pg/mL is predictive of MTC recurrence (19). However, the dynamics of preoperative normalization of serum Ctn were not well understood. PCT, a peptide precursor of Ctn, has been suggested as an excellent tumor marker for the follow-up of patients with MTC, while it is susceptible to systemic inflammatory response and stress activity (20). Additionally, Neoplastic C cells also produce CEA. There are evidences that the positivity of Ctn and CEA is related to a greater tumor burden and high levels of these two markers indicates the presence of metastatic disease (11, 12, 21). Moreover, Ctn doubling times is an excellent surveillance adjunct and can aid clinicians in predicting tumor recurrence (11, 12). Nigam et al. conducted the related research work and found that high-grade patients were associated with worse prognosis, especially when Ctn doubling times less than 2 years (13). However, the Ctn doubling time is calculated by following up with patients at least 4 times over 2years (3). While it is a good dynamic surveillance marker, calculating Ctn doubling time is quite time-consuming and may not reflect early clinical outcome after surgery. Of note, a basic research conducted by Baldini et al. offers a new perspective on MTC, revealing that three isoforms of Aurora kinase are expressed in MTC tissues and treatment of human medullary thyroid carcinoma derived cell line with an aurora kinase inhibitor leads to the decrease of proliferation and *in vitro* tumorogenicity (22). Future studies are required to explore the underlying mechanisms of MTC.

Few studies have investigated the role of postoperative Ctn for predicting prognosis in MTC. A recent study published an over two decades retrospective analysis showing that higher postoperative Ctn predicted both loco-regional recurrence/persistence and occurrence of distant metastases. However, the authors did not calculate an exact cut-off value of postoperative calcitonin for prognostic prediction (15). Moreover, Jung et al. suggested that clinicopathological characteristics and RFS of MTC improved over time and postoperative Ctn remission (<10 pg/ml) can predict disease recurrence. However, the authors used the same cut-off value of postoperative Ctn regardless of the change of measure methods over time (23). These studies indicate that postoperative Ctn has the equal significance with preoperative Ctn in reflecting the prognosis of patients with MTC.

In clinical work, we observed that the serum Ctn level in a large proportion of MTC patients could decrease to normal level within 1 week after surgery. Of note, Machens et al. also found that the status of lymph node metastasis and preoperative Ctn level could affect the normalization time of Ctn and Ctn levels typically normalize within 1 week (16). Moreover, we also found that there were several relevant researches reported that the early decrease of Ctn after surgery could evaluate the therapeutic effect and predict the prognosis (24–26). Cohen et al. showed that preoperative Ctn levels were predictor of postoperative Ctn normalization and primary tumor size, believing that the early normalization of Ctn after initial surgery was favorable for the outcomes of patients with MTC (27). In order to make a dynamic and accurate evaluation of the prognosis in patients with MTC, it is significant for clinicians to combine preoperative Ctn and postoperative Ctn comprehensively. We therefore hypothesized that the postoperative calcitonin-to-preoperative calcitonin ratio (CR), as a reflection of the degree of Ctn decline, can be used as a novel predictor of MTC.

In the present study, we investigated the role of CR for RFS in a cohort of 112 patients with sporadic MTC. Our study indicated that patients with higher CR were associated with greater risks of structural recurrence with a specific CR cut-off value of 0.125 and CR can predict long-term outcome in sporadic MTC patients. Remarkably, patients with CR≥0.125 had a larger proportion of persistent biochemical disease and structural disease. Among those patients with CR<0.125, 80.3% were disease-free, 7.4% were biochemical persistent disease, 11.1% progressed to structural disease and only 1.2% died of disease progression. In contrast, in patients with CR≥0.125, 22.6% progressed to disease-free status, 19.3% had a persistent biochemical disease, 48.4% had structural disease, and 9.7% died of disease progression at the final follow-up. Similar to our study, several previous studies which focused on the dynamic risk stratification of response to the initial therapy showed that excellent response patients were very likely to remain disease-free status and biochemical incomplete response patients were associated with a higher risk of persistent biochemical disease or structural disease at final follow-up (18, 28, 29). Nevertheless, there are minor differences with a higher proportion of disease-free patients in our study, probably because we adopted stricter exclusion criteria to exclude hereditary and distant metastases patients in order to better evaluate the efficacy of radical surgery. In addition, we used the optimal cut-off value of CR to define a high and low CR, which can eliminate the limitation of different Ctn testing assays over time, increasing the applicability and validity of CR value. Moreover, we observed that patients with CR≥0.125 were associated with more metastasized lymph nodes, extrathyroidal extension, higher T stage and advanced final disease status, meaning patients who have higher CR may be related to heavier disease burdens.

In our study, CR≥0.125 was a poor prognostic factor for RFS, and this association was maintained after an adjustment of the lowest AIC multivariate model with conventional risk factors. As a novel predictor of disease recurrence, the CR cut-off value of 0.125 was associated with a hazard ratio of 5.01, which was higher than conventional risk factors. The Kaplan-Meier survival analysis showed that the 5-year RFS rate of patients with CR≥0.125 was nearly 40% lower than those with patients with CR<0.125 (50.7% vs 90.3%), suggesting that patients who have higher CR should require greater scrutiny and closer follow-up after initial treatment. In addition to CR, metastasized lymph nodes≥10, multifocality and tumor size were also independent predictors for disease recurrence, indicating that more extensive lymph nodes metastasis and heavier tumor burden may largely reduce the likelihood of surgical cure. Previous studies have shown that tumor size, multifocal tumor, TNM stage, lymph nodes metastases, extrathyroidal extension, preoperative Ctn level and postoperative Ctn level are significantly associated with disease recurrence of MTC (14, 15, 30, 31). Our finding is consistent with those of most studies. While TNM stage and extrathyroidal extension were not independent factors in our study, these factors were valuable in the univariate analysis, which means patients with more advanced clinical stages might also be valued by clinicians.

As we all know, surgery is the main treatment modality for sporadic MTC. However, whether to perform total thyroidectomy for sporadic MTC with solitary tumor is still controversial. ATA guidelines regard total thyroidectomy as a standard surgical approach for patients with sporadic MTC (3), while unilateral thyroidectomy is recommended for sporadic MTC located only in one lobe by the Japan Associations of Endocrine Surgeons (JAES) (32). Compared with the multifocal characteristic of hereditary MTC, sporadic MTC tended to have a higher proportion of unifocality and better prognosis with a multifocal incidence of 16.0% (33). Whereas in our study, the proportion was as high as 27.7%, indicating that total thyroidectomy should be regard as the optimal initial treatment modality of sporadic MTC and reduce the incidence of failing to fully address the primary site of tumor. Over the past few decades, medical advances have improved the management of MTC. Dralle et al. revealed that there was a increasing number of patients with total thyroidectomy and decreasing number of patients with subtotal resection, while the incidence of vocal cord palsy and postoperative hypoparathyroidism had decreased over time (34). Similar phenomenon was also observed in our study. With the application of ultrasonic activated scalpel, bipolar electrocoagulation, carbon nanoparticle and intraoperative neuromonitoring of the recurrent laryngeal nerve, these improvements in instruments are helpful to shorten the learning curve of total thyroidectomy for surgeons. The main strength of our study is that the clinical management and follow-up of MTC patients occurred at a large tertiary center by a highly specialized team of experienced thyroid surgeons and oncologists.

Nomograms are progressively being used for estimating prognosis and personalized medicine, which can help clinicians to evaluate the prognosis of patients and apply the appropriate treatment (35). Based on CR and other independent risk factors, we established a novel prognostic model to predict the 3- and 5-year RFS for sporadic MTC. As a newly added factor in the nomogram, CR can offer a novel perspective for clinicians to assess the early postoperative status of patients and recognize a higher risk of disease recurrence more timely. Notably, the results of ROC and calibration showed that nomogram prediction has superior effective and predictive accuracy, compared to the classical TNM staging system. This nomogram, to a certain extent, could be used as a reference for predicting the prognosis of sporadic MTC patients and help clinicians to plan ongoing surveillance management during long-term follow-up.

There are some limitations in this study. First, the study was conducted retrospectively with a limited number of patients from a single center, making it susceptible to the inherent biases of such a study format. Therefore, future prospective multicenter studies are required to corroborate our findings. Second, a selection bias may occur in diagnosis and clinical treatment due to the relatively long period of data collection in this study. Third, we used the bootstrap resampling method for internal validation because of the relatively small sample size, but it is necessary to externally validate the performance of our model using other patient databases in the future.

## 5 Conclusions

In conclusion, CR is an independent predictor of structural recurrence in patients with sporadic MTC. The predictive nomogram based on CR appeared to be more accurate and reliable than the TNM staging system in predicting RFS for MTC patients after surgery, guiding clinicians to explain prognosis, predict cancer recurrence and develop individualize follow-up. Furthermore, the findings of our study can be readily translated to daily practice, and it would be interesting to determine the possible efficacy of CR in differentiating cases of persistence of dosable but clinically not significant postoperative Ctn values from those indicative of persistence of disease.

## Data availability statement

The raw data supporting the conclusions of this article will be made available by the authors, without undue reservation.

## Ethics statement

The studies involving human participants were reviewed and approved by The Medical Ethics Committee of Sun Yat-sen University Cancer Center. Written informed consent to participate in this study was provided by the participants’ legal guardian/next of kin.

## Author contributions

AY designed the main idea of the experiment. ZJ performed the main work of the research, and was a major contributor in writing the manuscript. All authors contributed to the article and approved the submitted version.
